# Novel epitopes identified from efflux pumps of *Mycobacterium tuberculosis* could induce cytotoxic T lymphocyte response

**DOI:** 10.7717/peerj.1229

**Published:** 2015-09-22

**Authors:** Ming-xia Zhai, Fei Chen, Yuan-yuan Zhao, Ya-hong Wu, Guo-dong Li, Yan-feng Gao, Yuan-ming Qi

**Affiliations:** School of Life Sciences, Zhengzhou University, Zhengzhou, China

**Keywords:** Cytotoxic T lymphocytes, Efflux pump, Epitope, Immunotherapy, *Mycobacterium tuberculosis*

## Abstract

Overcoming drug-resistance is one of the major challenges to control tuberculosis (TB). The up-regulation of efflux pumps is one common mechanism that leads to drug-resistance. Therefore, immunotherapy targeting these efflux pump antigens could be promising strategy to be combined with current chemotherapy. Considering that CD8+ cytotoxic T lymphocytes (CTLs) induced by antigenic peptides (epitopes) could elicit HLA-restricted anti-TB immune response, efflux pumps from classical ABC family (*Mycobacterium tuberculosis*, Mtb) were chosen as target antigens to identify CTL epitopes. HLA-A2 restricted candidate peptides from Rv2937, Rv2686c and Rv2687c of *Mycobacterium tuberculosis* were predicted, synthesized and tested. Five peptides could induce IFN-γ release and cytotoxic activity in PBMCs from HLA-A2^+^ PPD^+^ donors. Results from HLA-A2/K^b^ transgenic mice immunization assay suggested that four peptides Rv2937-p168, Rv2937-p266, Rv2686c-p151, and Rv2686c-p181 could induce significant CTL response *in vivo*. These results suggested that these novel epitopes could be used as immunotherapy candidates to TB drug-resistance.

## Introduction

Tuberculosis (TB) is a serious infection disease in developing countries, which is caused by the pathogen *Mycobacterium tuberculosis* (Mtb) (Dye & Williams, 2010). To date, the major strategies against tuberculosis are comprehensive chemotherapeutic regimens. However, with the emergence of multidrug-resistant tuberculosis (MDR-TB) and extensively drug resistant tuberculosis (XDR-TB), it becomes more difficult to fight against tuberculosis by chemotherapy alone. In addition to acquired drug resistance (Ramaswamy & Musser, 1998), intrinsic drug resistance has been proven to exist in *Mycobacterium tuberculosis* (De Rossi, Ainsa & Riccardi, 2006), in which active drug efflux pumps play important roles (Escribano et al., 2007; Spies et al., 2008).

Since Mtb is an intracellular pathogen living in macrophages, more and more evidence suggested the important role of cellular immunity in controlling its dissemination. It has been proven that, in addition to CD4^+^ T cells, MHC class I restricted CD8^+^ T cells also play very important roles in immune responses against Mtb. The role of CD8^+^ T cell-mediated cellular immunity was extensively described in Mtb-challenged mouse models, non-human primates, as well as patients (D’Souza et al., 2000; Flynn & Chan, 2001; Flynn et al., 1992; Lazarevic & Flynn, 2002; Sousa et al., 2000).

Although a lot of work has been done to develop new vaccines, BCG is the only approved one against tuberculosis, while its efficacy in adults has been questioned (Black et al., 2002). Considering all these reasons mentioned above, we believed that it would be very worthy to identify HLA-A2 restricted cytotoxic T lymphocyte (CTL) epitopes derived from drug efflux pump antigens of Mtb. It would help us to develop effective subunit vaccines against TB, especially XDR and MDR strains caused by over-expression of the efflux pumps.

Classical ABC (ATP binding cassette) family is the most well-known efflux pump family which is responsible for intrinsic drug resistance. It was shown that the over-expression of Rv2686c-Rv2687c-Rv2688c in *M. smegmatis* increased the minimum inhibitory concentrations of ciprofloxacin (Pasca et al., 2004). Rv2937 (drrB) together with drrA behaved as a functional efflux pump referring to drug resistance of rifampin, tetracycline and erythromycin (Choudhuri et al., 2002). Because of that, both drrA and Rv2688c have significant homology with some proteins in human, Rv2937, Rv2686c, and Rv2687c were chosen as target antigens to identify HLA-A2 restricted cytotoxic T lymphocyte epitopes.

Eight potential peptides derived from Rv2937, Rv2686c, and Rv2687c were predicted by using the online tools, SYFPEITHI, BIMAS, and NetCTL. These peptides were synthesized, and their ability to induce immune response was tested in PBMCs of HLA-A2^+^ donors (*in vitro*) and HLA-A2/K^b^ transgenic mice (*in vivo*).

## Materials and Methods

### Prediction and synthesis of candidate peptides

By using epitope prediction tools, BIMAS (http://bimas.dcrt.nih.gov/molbio/hla_bind/) (Parker, Bednarek & Coligan, 1994), SYFPEITHI (http://www.syfpeithi.de/Scripts/MHCServer.dll/EpitopePrediction.htm) (Rammensee et al., 1999), and NetCTL (http://www.cbs.dtu.dk/services/NetCTL/) (Larsen et al., 2007), potential HLA-A2-restricted T cell epitopes derived from Rv2937, Rv2686c, and Rv2687c were predicted. Peptides with relative high scores were synthesized by standard solid phase Fmoc strategy. The peptides were purified by reverse phase-high performance liquid chromatography (RP-HPLC), and their molecular weights were confirmed by electrospray ionization-mass spectrometry (ESI-MS). The peptide HBcAg_18–27_ (FLPSDFFPSV) was used as irrelevant negative control, and the HBV core antigen-derived T helper epitope (sequence128–140: TPPAYRPPNAPIL) was used to enhance the immune activity in the mice immunization experiment (Milich et al., 1988; Vissers et al., 1999).

### Blood samples, animals and cell lines

Whole blood samples were prepared from HLA-A2^+^ (PPD^+^ or PPD^−^) healthy donors. The HLA-A2.1/K^b^ transgenic mice were previously gifted by Professor Xue-tao Cao (Second Military Medical University) (Vitiello et al., 1991). All mice at 8 to 12 weeks in the experiments were housed in a specific pathogen-free environment in our laboratory. The sample collection from healthy donors and animal experiments were approved by the Ethics Committee of Zhengzhou University (No. 20120312). The human transporter associated with antigen processing (TAP) -deficient T2 cell line was kindly provided by professor Yu-zhang Wu (Third Military Medical University, China), and the cells were cultured in RPMI 1640 medium supplemented with 10% fetal bovine serum (FBS) and maintained at 37 °C in an incubator with a humidified atmosphere containing 5% CO_2_.

### Generation of CTLs from PBMCs of HLA-A2^+^ donors

The procedure of generation of CTLs *in vitro* was performed in accordance with the protocols described by our laboratory (Liu et al., 2012; Shi et al., 2013). Briefly, PBMCs were isolated from six HLA-A2^+^ PPD^+^ and an HLA-A2^+^ PPD^−^ healthy donors with centrifugation at a Ficoll-Paque density gradient and then cultured in IMDM medium supplemented with 10% FBS, 100 units/mL penicillin, and 100 µg/ml streptomycin under the condition of 37 °C, 5% CO_2_ (Han et al., 2006). Then, these cells were stimulated once a week with each candidate peptide (10 µg/ml) in the presence of 3 µg/ml *β*2-microglobulin. Human recombinant IL-2 (50 U/ml) and IL-7 (50 U/ml) were added both on day 3 and one day after each stimulation. Seven days after the third round of stimulation, the cytotoxic assay and ELISPOT assay were performed.

### Generation of CTLs from HLA-A2.1/K^b^ transgenic mice

CTLs from HLA-A2.1/K^b^ transgenic mice were generated as previously described (Liu et al., 2012; Shi et al., 2013). Briefly, HLA-A2.1/K^b^ transgenic mice were grouped randomly, and injected subcutaneously at the base of the tail with 100 µg each peptide emulsified in incomplete Freund’s adjuvant (IFA) in the presence of 140 µg of the T helper epitope every five days (Eguchi et al., 2006; Tourdot et al., 2000). On day 11, spleen lymphocytes of each mouse were separated and then re-stimulated with the corresponding peptide (10 µg/ml) *in vitro* for another five days. Then, the LDH cytotoxicity and ELISPOT assays were employed.

### ELISPOT assay

ELISPOT assay was performed according to the instruction of the commercial kit (Dakewe Biotech Company, Shenzen, China). Peptide-pulsed T2 cells (stimulator cells, 1 × 10^5^), along with the induced CTLs (effector cells, 1 × 10^5^), were seeded into an anti-human (or anti-mouse) IFN-*γ* antibody coated 96-well plate (Ding et al., 2009). After incubation for 16 h at 37 °C, cells were removed and plates were processed. Spots were counted with a computer-assisted spot analyzer (Dakewe Biotech Company, Shenzen, China).

### Cytotoxicity assay

Cytotoxic activity was tested by the non-radioactive cytotoxicity assay kit (Promega, US) at gradient E:T ratio according to the manufacturer’s instruction. T2 cells were loaded with 10 µg/ml peptide for 1 h at 37 °C as target cells. The effector cells were co-cultured with target cells (1 × 10^4^ cells/well) at various effector/target ratios for 5 h at 37 °C under 5% CO_2_. The percentage of specific lysis of the target cells was determined according to the following formula. Percentage of specific lysis = [(experimental release − effector spontaneous release − target spontaneous release)/(target maximum release − target spontaneous release)] × 100.

### Statistical analysis

All data were presented as means ± SD. Comparisons between experimental groups and relevant controls were analyzed by Student’s *t* test. *P* < 0.05 was considered as statistically significant difference.

## Results

### Peptides selected as potential CTL epitopes

By using the on-line prediction tools, eight potential HLA-A2 restricted CTL epitopes were selected from the three candidate efflux pump antigens: Rv2937, Rv2686c, and Rv2687c. These peptides were synthesized and their molecular weights were confirmed by ESI-MS. (Table 1).

**Table 1:** The data of ESI-MS and prediction of HLA-A2 restricted epitopes from efflux pumps of *Mycobacterium tuberculosis*.

Antigen	Position	Sequence	Scores			ESI-MS [M+H/Na]^+^	
			BIMAS	SYFPEITHI	NetCTL	Calculated	Observed
Rv2937	168	YIVGFCLLV	167.168	24	1.2629	1026.3	1026.7
	262	VMAPTLTWL	331.788	27	1.2098	1031.3	1031.7
	266	TLTWLFAFV	726.818	22	1.1149	1097.3	1097.7
Rv2686c	151	GLVAGLSAV	159.970	28	0.9644	786.0	786.7
	181	ALGMLIAGL	49.134	30	0.9898	858.1	858.7
	184	MLIAGLPCL	83.527	29	1.3358	930.2	930.6
Rv2687c	89	YLAAKLTVL	92.666	29	1.3019	991.2	991.8
	151	FLAAVIPLA	52.561	22	1.2362	914.2	914.6

### IFN-*γ* release ELISPOT assay *in vitro*

**Figure 1:**
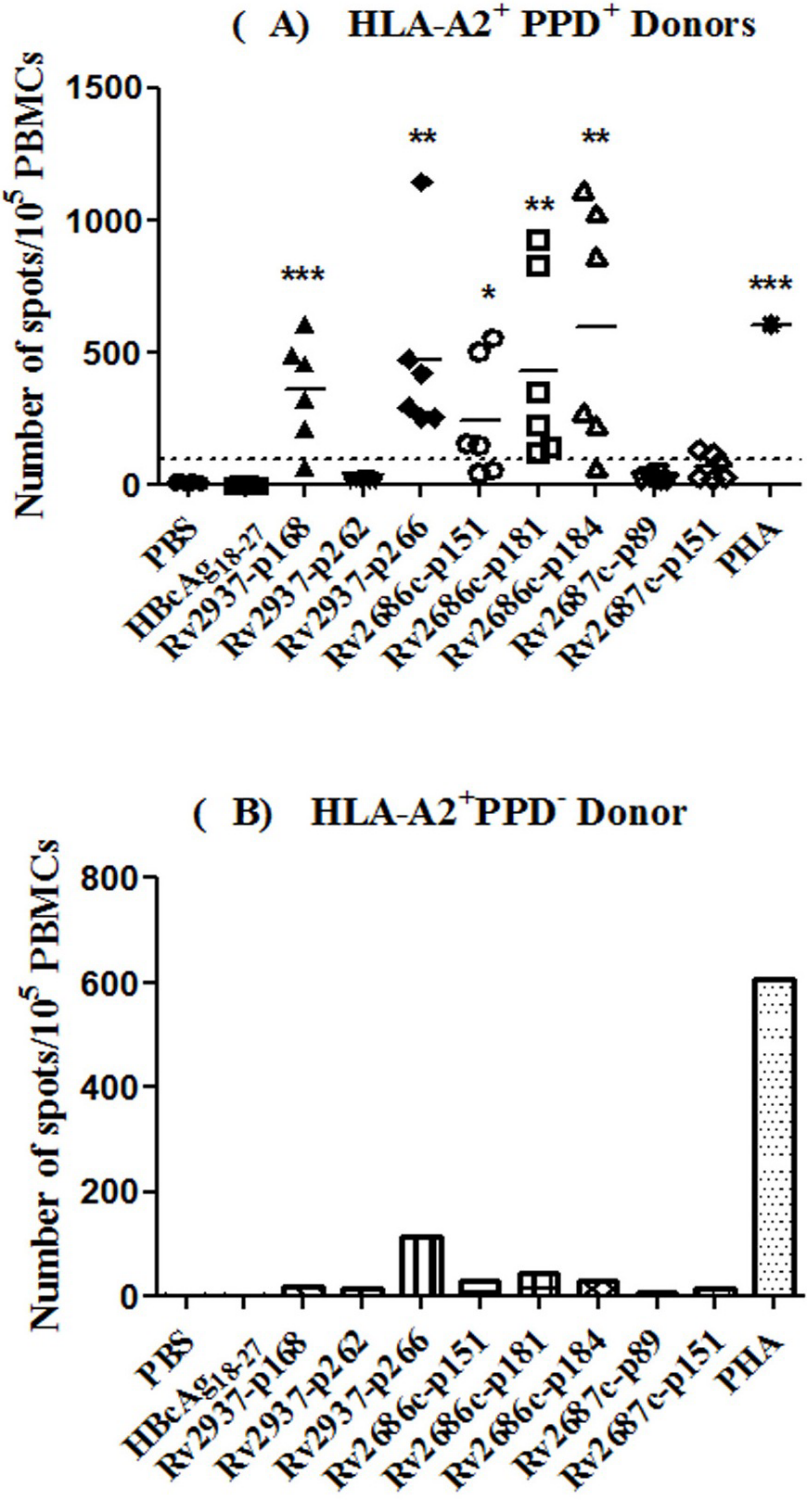
IFN-*γ* release assay by ELISPOT. Efflux pumps derived peptides were used to induce CTLs from PBMCs of (A) six HLA-A2^+^ PPD^+^ donors, and (B) one HLA-A2^+^ PPD^−^ donor *in vitro*. T2 cells incubated with corresponding peptide and irrelevant peptide (50 µg/ml) for 4 h were used as stimulators. PBMCs from different donors were separated and stimulated with synthetic peptides and IL-2 in IMDM supplemented with 10% FCS for 21 days. Then, these PBMCs were collected, and ELISPOT assay was performed to determine the IFN-*γ* production by these cells. CTLs induced by PBS and irrelevant peptide HBcAg_18–27_ were taken as negative controls. *P < 0.05, **P < 0.01, ***P < 0.001 represented the significances *vs* PBS group.

CTLs were induced from the PBMCs of six HLA − A2^+^ PPD^+^ and an HLA − A2^+^ PPD^−^ healthy donors. IFN-*γ* release ELISPOT assay was employed to test the capacity of the eight peptides to induce CTL response. As shown in Fig. 1A, Rv2937-p168, Rv2937-p266, Rv2686c-p151, Rv2686c-p181, and Rv2686c-p184, but not the irrelevant peptide HBcAg_18–27_, could induce more frequent and potent CTL response in the six HLA-A2^+^ PPD^+^ donors, while all these peptides could only induce very weak response in the HLA-A2^+^ PPD^−^ donor (Fig. 1B).

### *In vitro* cytotoxic activity of peptide-specific CTLs

**Figure 2:**
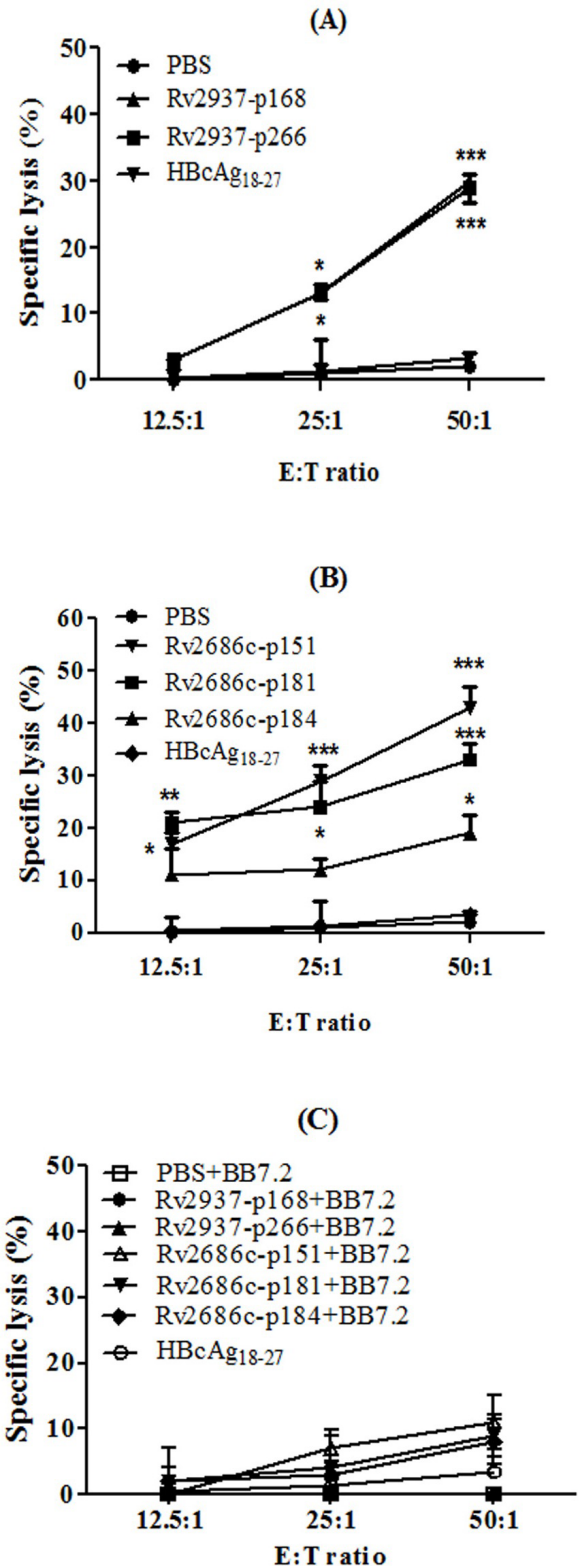
The cytotoxic activity of the CTLs induced by candidate peptides from human PBMCs. PBMCs from HLA-A2^+^ PPD^+^ donor were stimulated with each peptide (10 µg/mL) for three times. After 5 days of the final stimulation, the stimulated PBMCs were used as effector cells to detect their cytotoxic activity against target cells in LDH release assay. T2 cells loaded with peptide (HLA-A2^+^, antigen^+^), and T2 cells after which surface HLA-A2 molecules were blocked with anti-HLA-A2 monoclonal antibody (HLA-A2^−^, antigen^+^) were used as target cells. CTLs induced by PBS and irrelevant peptide HBcAg_18–27_ were taken as negative controls. (A–B) The effector cells were obtained from CTLs induced from PBMCs of HLA-A2^+^ PPD^+^ donors. (C) The HLA-A2 molecules on the T2 cells surface were blocked by anti-HLA-A2 monoclonal antibody. *P < 0.05, **P < 0.01, ***P < 0.001 represented the significances *vs* PBS group.

Based on the results of the ELISPOT assay, Rv2937-p168, Rv2937-p266, Rv2686c-p151, Rv2686c-p181, and Rv2686c-p184 were selected to investigate whether the CTLs they induced could lyse target cells. In the cytotoxicity assay, PBMCs from HLA-A2^+^ PPD^+^ donor were stimulated with synthetic peptides according to the previously described method for CTLs induction, peptide-pulsed T2 cells were considered as target cells (HLA-A2^+^, antigen^+^). As shown in Fig. 2, the specific lysis percentages of the CTLs induced by Rv2937-p168, Rv2937-p266, Rv2686c-p151, Rv2686c-p181, and Rv2686c-p184 from PBMCs of HLA-A2^+^ PPD^+^ healthy donor were increased along with the E/T ratio from 12.5:1 to 50:1. However, after incubating with anti-HLA-A2 monoclonal antibody, the specific lysis rates of the CTLs were greatly reduced. Meanwhile, CTLs induced by all these peptides in the HLA-A2^+^ PPD^−^ donor could not lyse the peptide-loaded T2 cells (Fig. S1). These results indicated that these peptides could induce HLA-A2-restricted CTL response in PPD^+^ healthy donor.

### Cytotoxic T lymphocyte response in HLA-A2.1/K^b^ transgenic mice

**Figure 3:**
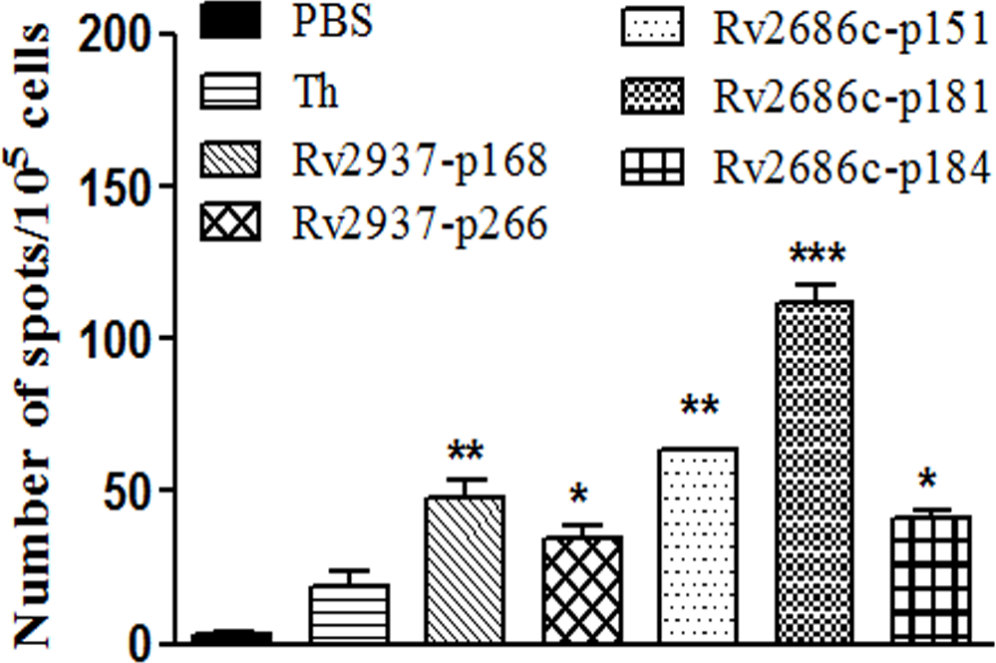
IFN-*γ* release ELISPOT assay of the spleen lymphocytes generated from immunized HLA-A2.1/K^b^ transgenic mice (*n* = 4). HLA-A2.1/K^b^ transgenic mice were immunized s.c with 100 µg each peptide emulsified in incomplete Freund’s adjuvant (IFA) in the presence of 140 µg of the T helper epitope on day 0, 5, and 10. Mice injected with IFA containing PBS or T helper epitope were used as negative control. On day 11, the animals were sacrificed; spleen lymphocytes were re-stimulated *in vitro* by the relevant peptides for another five days. T2 cells incubated with relevant peptides (50 µg/mL) for 4 h were used as stimulators. Each sample was measured in three replicates. *P < 0.05, **P < 0.01, ***P < 0.001 represented the significances *vs* Th epitope group.

HLA-A2.1/K^b^ transgenic mice immunization model has been widely used to study the *in vivo* CTL activity of HLA-A2-restricted epitopes. Since Rv2937-p168, Rv2937-p266, Rv2686c-p151, Rv2686c-p181, and Rv2686c-p184 could induce good immune response *in vitro*, we investigated whether these peptides could stimulate CTL response in HLA-A2.1/K^b^ transgenic mice. After immunization of each candidate peptide with Th epitope and IFA adjuvant, the spleen lymphocytes were isolated and stimulated with the corresponding peptide *in vitro* for another five days. IFN-*γ* release ELISPOT assay and LDH cytotoxicity assay were performed. As shown in Fig. 3, all of the five peptides showed more potent activity to induce IFN-*γ* release than that of the Th control group.

**Figure 4:**
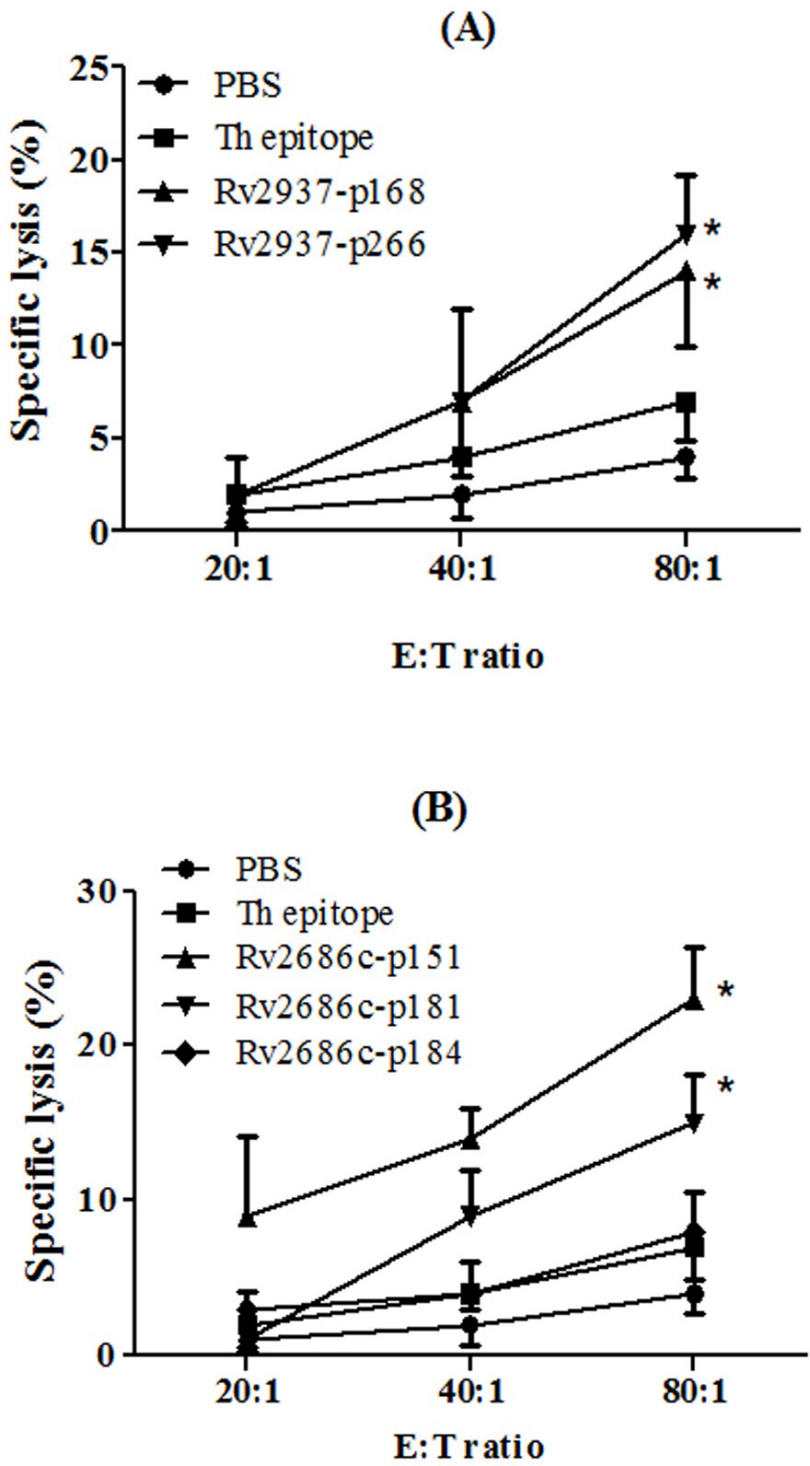
Specific lysis of T2 cells loaded with synthetic peptides by the CTLs generated from the immunized HLA-A2.1/K^b^ transgenic mice (*n* = 4). HLA-A2.1/K^b^ transgenic mice were immunized as described above. T2 cells incubated with relevant peptides (50 µg/mL) for 4 h were used as target cells. Each sample was measured in three replicates. *P < 0.05 represented the significances *vs* Th epitope group.

The cytotoxic activity of these spleen lymphocytes was also measured by LDH cytotoxicity assay. Peptide-loaded T2 cells were served as target cells and the effector/target ratios were 20:1, 40:1, and 80:1. As shown in Fig. 4, at the E:T ratio of 80:1, the CTLs induced by Rv2937-p168, Rv2937-p266, Rv2686c-p151, and Rv2686c-p181 could significantly kill the target cells. To our surprise, although Rv2686c-p184 could induce the most potent IFN-*γ* release activity in PBMCs of HLA-A2^+^ PPD^+^ donors, it could not induce CTLs with killing effects in HLA-A2.1/K^b^ transgenic mice.

## Discussion

Tuberculosis is considered to be a major public health concern worldwide because of its high morbidity and mortality rates. As reported, the emergence of XDR-TB and MDR-TB left patients with fewer options for treatment and at risk for higher mortalities, especially in HIV/TB co-infected patients (Principi & Esposito, 2015). At present, BCG has been proven to be the only available vaccine for TB prevention, but it could not protect adults in most cases. One reason for this is the BCG defects in some important antigens. There are no effective therapeutic vaccines for TB patients as well, especially for the drug-resistant ones. Development of novel therapeutic vaccines provides a promising strategy to be combined with chemotherapy. Accumulating data showed that subunit vaccine containing peptide or protein antigens could exhibit protective activity with very good safety (Ivanyi, 2014).

HLA-A2 is one of the most common supertypes of human leukocyte antigen particularly in Asian, with an estimated frequency of nearly 50% (Mehra et al., 2001). Cellular immunity mediated by CD8^+^ T cells is believed important to control the latent Mtb infection. Therefore, identification of HLA-A2 restricted cytotoxic T lymphocyte epitopes derived from efflux pump antigens could be helpful to develop vaccines against drug-resistant TB caused by intrinsic drug efflux. Recently, most researchers who work on the identification of CTL epitopes of Mtb antigens focus their work on secretary proteins, such as ESAT-6 (Lalvani et al., 1998), 19-kDa lipoprotein (Mohagheghpour et al., 1998), Ag85B (Geluk et al., 2000), 16-kDa antigen (Caccamo et al., 2002), MPT51 (Suzuki et al., 2004), and so on. We also identified such kind of epitopes from CFP21 and RD region antigens (Chen et al., 2012; Lv et al., 2010). Furthermore, we first reported that efflux pumps could also be considered as target antigens for TB immunotherapy, and found that Rv1410c could serve as a candidate to the vaccine design against drug-resistant Mtb (Zhu et al., 2011). We then screened classical efflux pump family members in the genome of TB to find more promising target antigens. As shown in the present study, we found that epitopes Rv2937-p168, Rv2937-p266, Rv2937-p168, Rv2686c-p181, and Rv2686c-p184 identified from ABC family members, could elicit good capacity to induce CTL response in HLA-A2^+^ PPD^+^ healthy donors and/or HLA-A2.1/K^b^ transgenic mice. Work still needs to be done to test whether infected macrophages could present the relevant peptides to CTLs, and to identify more promising antigens related to drug-resistant Mtb. We are now working on extrinsic drug-resistant antigens in Mtb. In the future, we hope we can combine all these epitopes derived from secretary and drug-resistant antigens to develop multi-valent subunit vaccines.

As is well known, early diagnosis of TB is fundamental for tuberculosis control. In the last decade, ELISPOT assay in the TB diagnosis is considered to have high specificity and sensitivity (Lalvani & Pareek, 2010; Milotic et al., 2011). Most of the five candidate peptides showed good activity among the six HLA-A2^+^ PPD^+^ healthy donors, but not in the HLA-A2^+^ PPD^−^ donor. Although we only tested the IFN-*γ* release assay in one HLA-A2^+^ PPD^−^ volunteer, and could not draw a conclusion from the results of our experiment, it is possible that these peptides have the potential to be used for TB diagnosis.

In the present study, we used so called ‘reversal immunology’ strategy to predict antigen epitopes by using on-line tools instead of time-consuming overlapping peptides method, which was proved to be very efficient. However, it may lead to large amounts of false positive and false negatives by using only one computational algorithm to predict CTL epitopes. Therefore, we used the epitope prediction tool NetCTL combined with the widely used BIMAS and SYFPEITHI databases. NetCTL integrates prediction of binding affinity, transporter of antigenic peptide efficiency and proteasomal cleavage (Larsen et al., 2007), SYFPEITHI is a motif-matrix-based prediction method for MHC binding prediction (Rammensee et al., 1999) and BIMAS is based on peptide/MHC complex half-life (Parker, Bednarek & Coligan, 1994). Our results suggested that this strategy could be very efficient and successful.

## Conclusions

In conclusion, we have identified five HLA-A2 restricted cytotoxic T lymphocyte epitopes derived from drug efflux pump antigens of *Mycobacterium tuberculosis*. The epitopes Rv2937-p168, Rv2937-p266, Rv2686c-p151, Rv2686c-p181, and Rv2686c-p184 showed moderate capacity to induce CTL response in HLA-A2^+^ PPD^+^ donors. Except for Rv2686c-p184, other epitopes could also elicit CTL response when immunized in HLA-A2.1/K^b^ transgenic mice. These epitopes could serve as candidates for TB subunit vaccines development.

## Supplemental Information

10.7717/peerj.1229/supp-1 Data S1 Raw data

10.7717/peerj.1229/supp-2 Supplemental Information 1 Data of CTL lysis activity from PPD-donor

## References

[ref-1] Black GF, Weir RE, Floyd S, Bliss L, Warndorff DK, Crampin AC, Ngwira B, Sichali L, Nazareth B, Blackwell JM, Branson K, Chaguluka SD, Donovan L, Jarman E, King E, Fine PE, Dockrell HM (2002). BCG-induced increase in interferon-gamma response to mycobacterial antigens and efficacy of BCG vaccination in Malawi and the UK: two randomised controlled studies. Lancet.

[ref-2] Caccamo N, Milano S, Di Sano C, Cigna D, Ivanyi J, Krensky AM, Dieli F, Salerno A (2002). Identification of epitopes of *Mycobacterium tuberculosis* 16-kDa protein recognized by human leukocyte antigen-A*0201 CD8(+) T lymphocytes. Journal of Infectious Diseases.

[ref-3] Chen F, Zhai MX, Zhu YH, Qi YM, Zhai WJ, Gao YF (2012). *In vitro* and *in vivo* identification of a novel cytotoxic T lymphocyte epitope from Rv3425 of *Mycobacterium tuberculosis*. Microbiology and Immunology.

[ref-4] Choudhuri BS, Bhakta S, Barik R, Basu J, Kundu M, Chakrabarti P (2002). Overexpression and functional characterization of an ABC (ATP-binding cassette) transporter encoded by the genes drrA and drrB of *Mycobacterium tuberculosis*. Biochemical Journal.

[ref-5] D’Souza CD, Cooper AM, Frank AA, Ehlers S, Turner J, Bendelac A, Orme IM (2000). A novel nonclassic beta2-microglobulin-restricted mechanism influencing early lymphocyte accumulation and subsequent resistance to tuberculosis in the lung. American Journal of Respiratory Cell and Molecular Biology.

[ref-6] De Rossi E, Ainsa JA, Riccardi G (2006). Role of mycobacterial efflux transporters in drug resistance: an unresolved question. FEMS Microbiology Reviews.

[ref-7] Ding FX, Wang F, Lu YM, Li K, Wang KH, He XW, Sun SH (2009). Multiepitope peptide-loaded virus-like particles as a vaccine against hepatitis B virus-related hepatocellular carcinoma. Hepatology.

[ref-8] Dye C, Williams BG (2010). The population dynamics and control of tuberculosis. Science.

[ref-9] Eguchi J, Hatano M, Nishimura F, Zhu X, Dusak JE, Sato H, Pollack IF, Storkus WJ, Okada H (2006). Identification of interleukin-13 receptor alpha2 peptide analogues capable of inducing improved antiglioma CTL responses. Cancer Research.

[ref-10] Escribano I, Rodriguez JC, Llorca B, Garcia-Pachon E, Ruiz M, Royo G (2007). Importance of the efflux pump systems in the resistance of *Mycobacterium tuberculosis* to fluoroquinolones and linezolid. Chemotherapy.

[ref-11] Flynn JL, Chan J (2001). Immunology of tuberculosis. Annual Review of Immunology.

[ref-12] Flynn JL, Goldstein MM, Triebold KJ, Koller B, Bloom BR (1992). Major histocompatibility complex class I-restricted T cells are required for resistance to *Mycobacterium tuberculosis* infection. Proceedings of the National Academy of Sciences of the United States of America.

[ref-13] Geluk A, Van Meijgaarden KE, Franken KL, Drijfhout JW, D’Souza S, Necker A, Huygen K, Ottenhoff TH (2000). Identification of major epitopes of *Mycobacterium tuberculosis* AG85B that are recognized by HLA-A*0201-restricted CD8+ T cells in HLA-transgenic mice and humans. Journal of Immunology.

[ref-14] Han JF, Zhao TT, Liu HL, Lin ZH, Wang HM, Ruan ZH, Zou LY, Wu YZ (2006). Identification of a new HLA-A*0201-restricted cytotoxic T lymphocyte epitope from CML28. Cancer Immunology and Immunotherapy.

[ref-15] Ivanyi J (2014). Function and potentials of *M. tuberculosis* epitopes. Frontiers in Immunology.

[ref-16] Lalvani A, Brookes R, Wilkinson RJ, Malin AS, Pathan AA, Andersen P, Dockrell H, Pasvol G, Hill AV (1998). Human cytolytic and interferon gamma-secreting CD8+ T lymphocytes specific for *Mycobacterium tuberculosis*. Proceedings of the National Academy of Sciences of the United States of America.

[ref-17] Lalvani A, Pareek M (2010). Interferon gamma release assays: principles and practice. Enfermedades Infecciosas Y Microbiologia Clinica.

[ref-18] Larsen MV, Lundegaard C, Lamberth K, Buus S, Lund O, Nielsen M (2007). Large-scale validation of methods for cytotoxic T-lymphocyte epitope prediction. BMC Bioinformatics.

[ref-19] Lazarevic V, Flynn J (2002). CD8+ T cells in tuberculosis. American Journal of Respiratory and Critical Care Medicine.

[ref-20] Liu W, Zhai M, Wu Z, Qi Y, Wu Y, Dai C, Sun M, Li L, Gao Y (2012). Identification of a novel HLA-A2-restricted cytotoxic T lymphocyte epitope from cancer-testis antigen PLAC1 in breast cancer. Amino Acids.

[ref-21] Lv H, Gao Y, Wu Y, Zhai M, Li L, Zhu Y, Liu W, Wu Z, Chen F, Qi Y (2010). Identification of a novel cytotoxic T lymphocyte epitope from CFP21, a secreted protein of *Mycobacterium tuberculosis*. Immunology Letters.

[ref-22] Mehra NK, Jaini R, Rajalingam R, Balamurugan A, Kaur G (2001). Molecular diversity of HLA-A*02 in Asian Indians: predominance of A*0211. Tissue Antigens.

[ref-23] Milich DR, Hughes JL, McLachlan A, Thornton GB, Moriarty A (1988). Hepatitis B synthetic immunogen comprised of nucleocapsid T-cell sites and an envelope B-cell epitope. Proceedings of the National Academy of Sciences of the United States of America.

[ref-24] Milotic DM, Popovic-Grle S, Katalinic-Jankovic V, Simunovic A (2011). Comparison of new and old tests for the diagnosis of latent tuberculosis infection (quantiferon and TST). Lijecnicki Vjesnik.

[ref-25] Mohagheghpour N, Gammon D, Kawamura LM, Van Vollenhoven A, Benike CJ, Engleman EG (1998). CTL response to *Mycobacterium tuberculosis*: identification of an immunogenic epitope in the 19-kDa lipoprotein. Journal of Immunology.

[ref-26] Parker KC, Bednarek MA, Coligan JE (1994). Scheme for ranking potential HLA-A2 binding peptides based on independent binding of individual peptide side-chains. Journal of Immunology.

[ref-27] Pasca MR, Guglierame P, Arcesi F, Bellinzoni M, De Rossi E, Riccardi G (2004). Rv2686c-Rv2687c-Rv2688c, an ABC fluoroquinolone efflux pump in *Mycobacterium tuberculosis*. Antimicrobial Agents and Chemotherapy.

[ref-28] Principi N, Esposito S (2015). The present and future of tuberculosis vaccinations. Tuberculosis (Edinb).

[ref-29] Ramaswamy S, Musser JM (1998). Molecular genetic basis of antimicrobial agent resistance in *Mycobacterium tuberculosis*: 1998 update. Tubercle and Lung Disease.

[ref-30] Rammensee H, Bachmann J, Emmerich NP, Bachor OA, Stevanovic S (1999). SYFPEITHI: database for MHC ligands and peptide motifs. Immunogenetics.

[ref-31] Shi RR, Liu J, Zou Z, Qi YM, Zhai MX, Zhai WJ, Gao YF (2013). The immunogenicity of a novel cytotoxic T lymphocyte epitope from tumor antigen PL2L60 could be enhanced by 4-chlorophenylalanine substitution at position 1. Cancer Immunology and Immunotherapy.

[ref-32] Sousa AO, Mazzaccaro RJ, Russell RG, Lee FK, Turner OC, Hong S, Van Kaer L, Bloom BR (2000). Relative contributions of distinct MHC class I-dependent cell populations in protection to tuberculosis infection in mice. Proceedings of the National Academy of Sciences of the United States of America.

[ref-33] Spies FS, Da Silva PE, Ribeiro MO, Rossetti ML, Zaha A (2008). Identification of mutations related to streptomycin resistance in clinical isolates of *Mycobacterium tuberculosis* and possible involvement of efflux mechanism. Antimicrobial Agents and Chemotherapy.

[ref-34] Suzuki M, Aoshi T, Nagata T, Koide Y (2004). Identification of murine H2-Dd- and H2-Ab-restricted T-cell epitopes on a novel protective antigen, MPT51, of *Mycobacterium tuberculosis*. Infection and Immunity.

[ref-35] Tourdot S, Scardino A, Saloustrou E, Gross DA, Pascolo S, Cordopatis P, Lemonnier FA, Kosmatopoulos K (2000). A general strategy to enhance immunogenicity of low-affinity HLA-A2. 1-associated peptides: implication in the identification of cryptic tumor epitopes. European Journal of Immunology.

[ref-36] Vissers JL, De Vries IJ, Schreurs MW, Engelen LP, Oosterwijk E, Figdor CG, Adema GJ (1999). The renal cell carcinoma-associated antigen G250 encodes a human leukocyte antigen (HLA)-A2.1-restricted epitope recognized by cytotoxic T lymphocytes. Cancer Research.

[ref-37] Vitiello A, Marchesini D, Furze J, Sherman LA, Chesnut RW (1991). Analysis of the HLA-restricted influenza-specific cytotoxic T lymphocyte response in transgenic mice carrying a chimeric human-mouse class I major histocompatibility complex. Journal of Experimetnal Medicine.

[ref-38] Zhu YH, Gao YF, Chen F, Liu W, Zhai MX, Zhai WJ, Qi YM, Ye Y (2011). Identification of novel T cell epitopes from efflux pumps of *Mycobacterium tuberculosis*. Immunology Letters.

