# Supplementary material for: Novel epitopes identified from efflux pumps of Mycobacterium tuberculosis could induce cytotoxic T lymphocyte response
Source: PeerJ. 2015 Sep 22;3:e1229. doi: 10.7717/peerj.1229 (PMC4582945; doi:10.7717/peerj.1229)
Supplement: Supplemental Information 1 [file peerj-03-1229-s002.pdf]

(A)

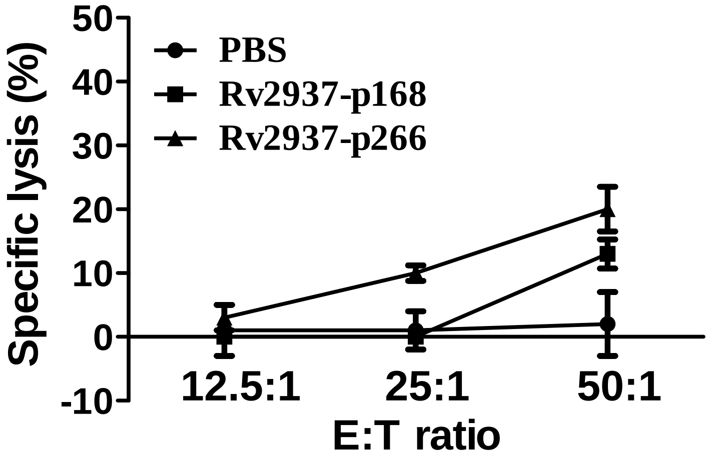

(B)

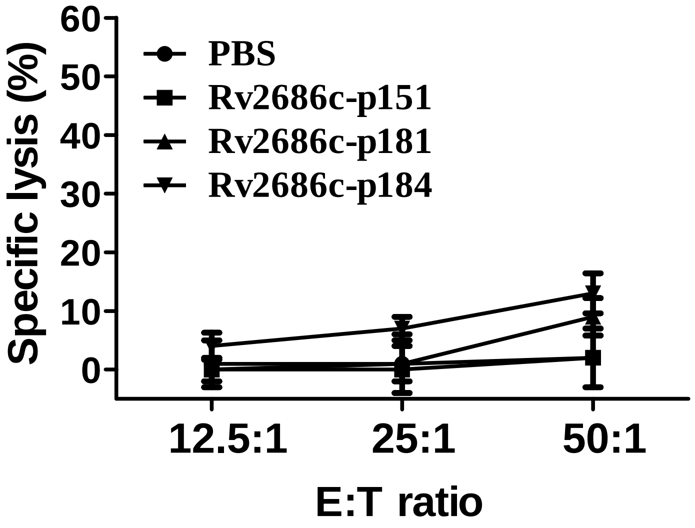

Figure S1. Specific lysis of T2 cells incubated with synthetic peptide by the CTLs generated from PBMCs of HLA-A*02^{+}PPD^{-} donor.
